# Robotic-assisted vs. fluoroscopy-assisted MIS-TLIF: improved screw accuracy and reduced early opioid use with comparable long-term outcomes

**DOI:** 10.3389/fsurg.2026.1837265

**Published:** 2026-06-30

**Authors:** Daniel W. Griepp, Bryce Sarcar, Hepzibha Alexander, Rabia Ahmed, Andrew Beggs, Armando Bunjaj, Jeffrey P. Turnbull, Joshua Caskey, Shivum Desai, Heather Heitkotter, Daniel A. Carr

**Affiliations:** 1 Department of Neurosurgery, Henry Ford Health Providence Hospital, Michigan State University College of Human Medicine, Southfield, MI, United States; 2 Department of Medical Education, Lake Erie College of Medicine, Erie, PA, United States

**Keywords:** inpatient opioid consumption, minimally invasive surgery, morphine milligram equivalents, patient reported outcomes (PROs), pedicle screw accuracy, robotic assisted surgery, spine surgery, transforaminal lumbar interbody fusion (TLIF)

## Abstract

**Introduction:**

Robotic-assisted (RA) techniques are increasingly used in minimally invasive transforaminal lumbar interbody fusion (MIS-TLIF). While technical advantages of RA have been demonstrated, it remains unclear whether they translate into improved patient-reported outcomes (PROs) or reduced opioid utilization compared to conventional fluoroscopy-assisted (FA) techniques. Given the growing emphasis on opioid stewardship in spine surgery, understanding differences in perioperative and long-term opioid use is critical.

**Methods:**

A retrospective cohort study was conducted using a database of patients who underwent single-level MIS-TLIF for degenerative lumbar pathology by a single surgeon between 2019 and 2024. Patients were grouped based on pedicle screw placement technique (RA vs. FA). Primary outcomes included inpatient opioid consumption measured in morphine milligram equivalents (MME), long-term opioid use at 3, 6, and 12 months, and PROs including Oswestry Disability Index (ODI) and Visual Analog Scale (VAS) scores. Secondary outcomes included operative time, length of stay (LOS), complications, and pedicle screw accuracy assessed by the Gertzbein and Robbins classification.

**Results:**

A total of 87 patients met the inclusion criteria (FA: *n* = 39; RA: *n* = 48), with no significant differences in baseline characteristics. Operative time, anesthesia time, LOS, and complication rates were similar between groups. The RA group demonstrated significantly improved screw accuracy, with a higher proportion of grade A screws (91% vs. 57%, *p* < 0.001). Inpatient MME was significantly lower in the RA group on postoperative days 0–1 (*p* = 0.002) and across total LOS (*p* = 0.0015). After adjusting for age, BMI, and opioid naïve status, the RA approach remained independently associated with lower opioid use on postoperative days 0–1 (*β* = −37.1; 95% CI, −63.6 to −10.6; *p* = 0.007) and total LOS (*β* = −17.1; 95% CI, −29.6 to −4.7; *p* = 0.008). Long-term opioid use at 3, 6, and 12 months was similar between groups (*p* > 0.05). Both groups demonstrated significant improvement in ODI and VAS scores at 6 and 12 months (*p* < 0.05), with no between-group differences in PRO improvement or minimal clinically important difference or patient acceptable symptom state achievement.

**Conclusion:**

RA MIS-TLIF had improved pedicle screw accuracy and reduced early postoperative opioid consumption compared to FA techniques; however, these advantages did not translate into reduced long-term opioid use or improved patient-reported outcomes.

## 1 Introduction

Low back pain is the most common musculoskeletal condition worldwide and a leading cause of disability, a burden that is expected to grow with an aging population (1–3). While most cases are managed conservatively, surgery is indicated for patients with refractory symptoms or neurologic decline (4). Transforaminal lumbar interbody fusion (TLIF), performed via open or minimally invasive (MIS) approaches, is a widely used treatment for degenerative lumbar disease, with increasing adoption of robotic-assisted techniques for pedicle screw placement (5–9). Alongside surgical advances, reducing perioperative opioid use has become a priority, as lumbar fusion patients are at risk for prolonged use and associated complications (10–14).

At our institution, one surgeon's transition from fluoroscopy-assisted (FA) to robotic-assisted (RA) screw placement following the adoption of a Mazor X Stealth system in 2022 created an opportunity to compare techniques within a single-surgeon cohort. This study evaluates differences in opioid use, patient-reported outcomes, and screw accuracy between approaches in single-level MIS-TLIF, with the hypothesis that outcomes would be comparable.

## 2 Materials and methods

### 2.1 Study design and setting

This manuscript was reported following the STROBE statement for cohort studies (15). Following Institutional Review Board approval (RMI20230071), a retrospective review of a prospectively maintained database at a single institution was performed for consecutive patients who underwent RA MIS-TLIF between February 2022 and December 2024 for degenerative lumbar spine pathologies. To minimize variability and strengthen internal consistency, we included only elective, single-level MIS-TLIF surgeries performed by a single fellowship-trained neurosurgeon, who is proficient and experienced in both RA and FA techniques (D.A.C.). All RA surgeries were performed using the third generation Mazor robotic system (Mazor X Stealth, Mazor Robotics Ltd, Caesarea, Israel). A cohort of patients who underwent FA single-level MIS-TLIF primarily between November 2019 and December 2022 by the same surgeon served as the comparator group.

Patients were excluded if (1) the indication for surgery was trauma or fracture; (2) pedicle screws were placed with midline incision, open exposure, or non-robotic Stealth navigation; (3) underlying malignancy or known spinal metastatic lesions; (4) osteomyelitis or osteodiscitis; (5) patient pathology requiring multiple segment fusions; (6) constructs including thoracic vertebral segments and/or pelvic fixation; (7) postoperative computed tomography (CT) scan was not performed; (8) follow up PRO data was not available; or (9) opioid use was unable to be obtained for at least 6 months postoperatively. Patients with revision surgeries were included and defined as having existing hardware, either with fusion of the same level or extension fusion above or below the construct, requiring replacement or revision of existing hardware.

### 2.2 Patient characteristics

Baseline patient demographics and comorbidities, including age, sex, smoking history, diabetes mellitus, preoperative opioid use, and body mass index (BMI) were collected through review of the electronic medical records (EMR). All patients underwent posterior instrumentation with four total pedicle screws and single interbody fusion with cage and rod fixation. During the transition period in 2022, the decision to place pedicle screws using either the FA or RA technique was based on the senior authors preference. Following surgery, all patients were administered a standardized multimodal pain management protocol that was unchanged throughout the study period. This included intravenous hydromorphone 0.5–2 mg every 3 h as needed for severe pain, two tablets of oral oxycodone 5 mg every 4 h as needed for moderate pain, in addition to acetaminophen as needed for mild pain and scheduled oral methocarbamol 750 mg three times daily. Collected operative variables were surgical indication, presence of concomitant decompression, length of stay (LOS), and any medical or surgical complications. The operative day (day 0) was included as 1 day in LOS; thus, a patient discharged on postoperative day (POD) 1 was considered to have a 2-day LOS.

### 2.3 Surgical technique

The surgical technique used by the senior author (D.A.C) was standardized in all cases. Patients are positioned prone on a Jackson table and prepped and draped in a standard sterile fashion. Prophylactic antibiotics are given.

In RA cases, the Mazor X device is mounted to the Jackson table, attached to the patient with a posterior superior iliac spine pin, and registered to a pre-operative CT with anteroposterior and oblique fluoroscopy. Paramedian incisions are precisely marked using the robotic arm projection. A sequence of drill, tap, and screw (Voyager, Medtronic) is used to place hardware on the contralateral side to the facetectomy and Kirschner wires on the ipsilateral side. On the contralateral side, trajectories are drilled into the facet joint and decorticated, followed by placement of allograft for posterolateral fusion. In FA cases, bilateral paramedian skin incisions are made lateral to the lateral pedicle line using estimation guided by biplanar fluoroscopy. The Jamshidi needle is then used to cannulate the pedicles down to the mid-vertebral body. Kirschner wires are placed and secured to the patient.

All decompressions and interbody placement are performed through the same paramedian incision using tubular dilators (MetRx, Medtronic). Standard facetectomy and laminectomy (ipsilateral and occasionally contralateral) are performed through a percutaneous MIS tube. The decision to use expandable vs. static interbody is made intraoperatively. After pedicle screw insertion, rods are measured, passed, and secured with set screws at the manufacturer's recommended torque. Final intraoperative anteroposterior and lateral radiographs were used to confirm hardware positioning in both the RA and FA groups before skin closure.

### 2.4 Outcome measurements

The primary outcomes of interest were inpatient postoperative opioid consumption measured in intravenous morphine milliequivalent (MME), PROs (ODI and VAS back and leg), and long-term postoperative opioid use. Inpatient MME was calculated using the medication administration record summary in the EMR for POD 0–1, POD 2 (if still admitted), and the overall daily mean for the total LOS. Patients were classified as non-opioid naïve if they used opioids for at least 7 days in the 30 days, leading to surgery. Long term postoperative opioid use was determined by whether each patient had opioid prescriptions dispensed at 3-, 6-, and 12-month intervals following surgery. Secondary outcomes included operative time, anesthesia time, intraoperative fluoroscopy dose, LOS, screw accuracy, and complications. Screw accuracy was determined by the Gertzbein and Robbins classification system (16). Grades A and B were considered acceptable, while grades C-E were considered unacceptable. Complications were characterized as deep vein thrombosis or pulmonary embolism within 90 days of surgery, pneumonia or sepsis within 30 days, large vessel injury, retroperitoneal hematoma, ileus or uninary retention, visceral injury, and the need for revision surgery due to screw malposition, wound infection, or other causes.

### 2.5 Statistical analysis

Patient demographics, along with perioperative and operative variables, were compared between the RA and FA groups using Fisher's exact test for nominal variables and unpaired *t*-tests for continuous variables. Screw accuracy and postoperative opioid use were analyzed using the Chi-square test. Fisher's exact test was used to compare the number of cases with excellent and acceptable screw placement between groups, as well as the number of patients who were opioid-naive before surgery. Longitudinal changes in PROs within the same patient were assessed using paired *t*-tests and completed using IBM SPSS Statistics (v29.0.2.0) for Windows (Chicago, IL). Changes in postoperative PROs at 6- and 12-months were analyzed between the RA and FA groups using independent samples *t*-tests. Fisher's exact test was used to compare the number of patients in the RA and FA groups with a change in PROs (baseline to 6- and 12-months postop) that exceeded the Minimal Clinically Important Difference (MCID) or met the patient acceptable symptom state (PASS). The MCID and PASS cutoff values (ODI: MCID Δ10, PASS ≤25 and VAS: MCID Δ1, PASS ≤3.3) have been published previously for the PROs measured in this study (17–19). The difference in average patient satisfaction between groups was analyzed using a Mann–Whitney *U* test.

Multivariable linear regression models were used to assess the independent effect of baseline PROs and surgical approach on postoperative inpatient MME consumption, with adjustment for age, BMI, and opioid naïve status. Multivariable linear regression was also used to evaluate the association between surgical approach and PROs at 6 and 12 months, adjusting for the corresponding preoperative score. Multivariable logistic regression was performed to assess predictors of achieving MCID for PROs. Unless otherwise stated, these analyses were completed using Microsoft Excel and GraphPad Prism (v10.4.1) for Windows (Boston, MA). *P*-values less than 0.05 were statistically significant.

## 3 Results

**Figure 1:**
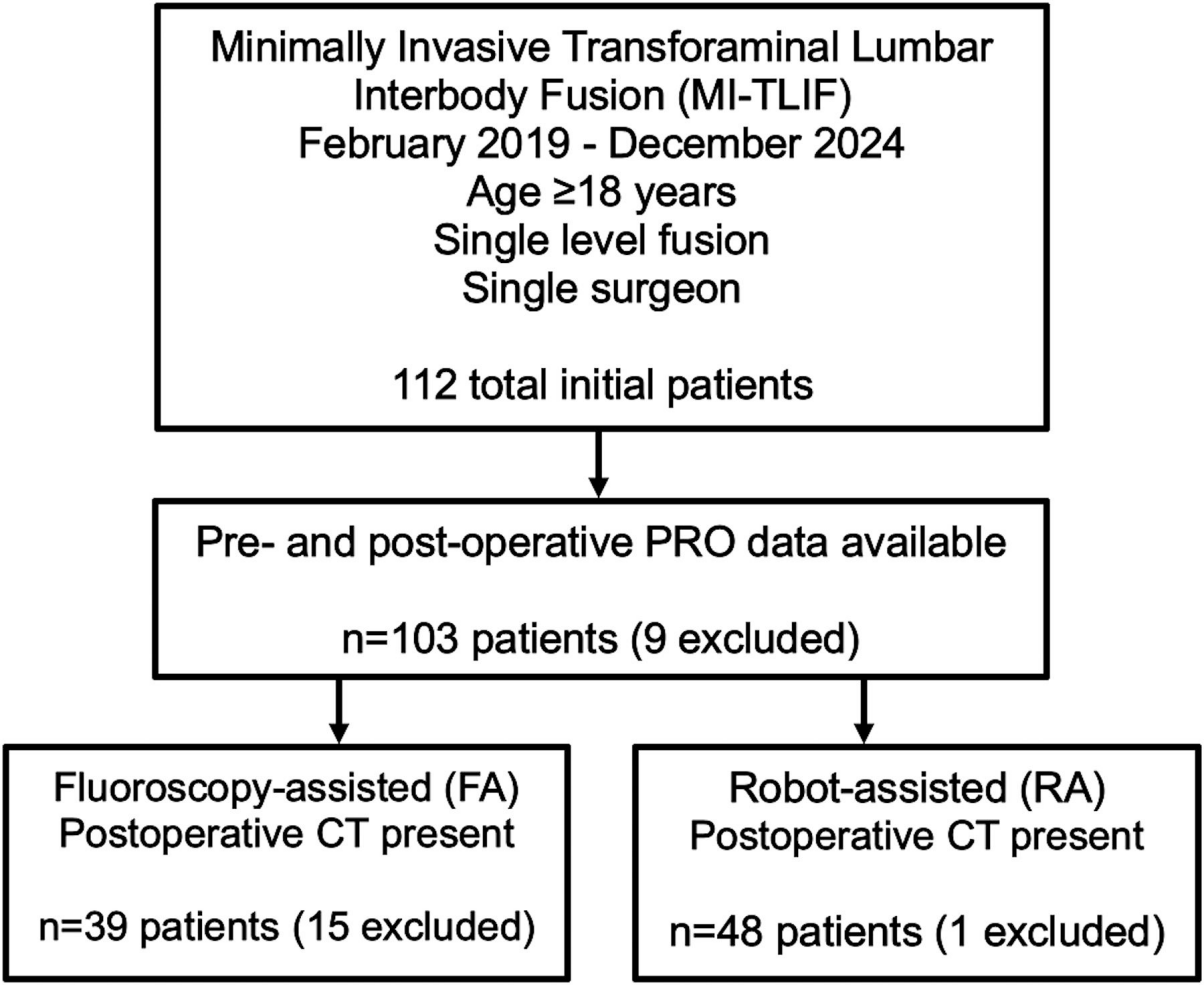
STROBE diagram.

A total of 112 patients underwent single-level MIS-TLIF by a single surgeon during the study period, of whom 87 (27 men and 60 women) satisfied the inclusion criteria. The STROBE diagram is shown in Figure 1. Thirty-nine patients (44.8%) underwent FA, and 48 (55.2%) underwent RA pedicle screw placement. Only FA cases were performed from November 2019 to January 2022, while February to December 2022 represented a mix of both FA and RA approaches, prior to the universal adoption of RA approach in January 2023. After this time, only three patients underwent FA approach due to downtime maintenance of the Mazor robotic system that prevented its use.

There was no significant difference in baseline characteristics between FA and RA cases for age, sex, BMI, tobacco use, opioid use before surgery, or history of diabetes (Table 1). The number of patients who had a prior decompression at the same level and/or a prior fusion at an adjacent level was not significantly different between groups (FA group 8/39 vs. RA group 9/48).

**Table 1:** Patient demographics.

*N* = 87	Fluoroscopy-assisted (FA) (*n* = 39)	Robot-assisted (RA) (*n* = 48)	*p*-value
Age	53.90 ± 13.63	58.35 ± 12.49	0.12
Male sex	13 (33.3)	14 (29.2)	0.82
Body mass index	35.09 ± 9.17	31.34 ± 6.11	0.057
Tobacco use	11 (28.2)	11 (22.9)	0.63
Diabetes	9 (23.1)	12 (25.0)	>0.99
Opioid naive	22 (56.4)	31 (64.6)	0.51
Prior lumbar surgery	8 (21.0)	9 (19.0)	>0.99
Primary surgical indication			
Degenerative disc disease	19 (48.7)	25 (52.1)	0.75
Degenerative spondylolisthesis	11 (28.2)	13 (27.1)	0.91
Adjacent segment disease	4 (10.3)	2 (4.2)	0.26
Recurrent disc herniation	2 (5.1)	6 (12.5)	0.24
Isthmic spondylolisthesis	3 (7.7)	2 (4.2)	0.48

Nominal data are presented as the number cases, *n* (%) that include “yes”; continuous data are presented as mean ± SD; age is measured in years; *p*-values <0.05 are considered significant.

### 3.1 Perioperative outcomes

Operative time, anesthesia time, and length of stay were similar in the FA and RA groups. Intraoperative fluoroscopy dose was significantly higher in the FA group (104 ± 82 mGy) compared to the RA group (15.3 ± 10 mGy, *p* < 0.001). The number of perioperative complications between the FA (6, 15.4%) and RA (5, 10.4%) groups was similar. Fifty-six (64.4%) patients were discharged home on POD 1, and 70 (80.4%) were discharged home on or by POD 2. LOS and/or need for rehabilitation did not differ significantly between FA and RA groups. This is further listed in Table 2. In the FA group, these included wound infection (1), sepsis (1), blood transfusion (1), compressive hematoma requiring return to operating room (2), and acute radiculopathy secondary to mispositioned screw (1). Complications in the RA group included urinary retention (2), ileus (1), pneumonia (1), and compressive hematoma requiring return to the operating room (1).

**Table 2:** Operative and perioperative clinical outcomes.

*N* = 87	Fluoroscopy-assisted (FA) (*n* = 39)	Robot-assisted (RA) (*n* = 48)	*p*-value
Operative time (min)	124 ± 30	129 ± 28	0.42
Anesthesia time (min)	182 ± 32	190 ± 31	0.24
Intraoperative fluoroscopy dose (mGy)	104 ± 82	15.3 ± 10	<0.001
LOS (days)	2.82 ± 1.52	2.69 ± 1.34	0.66
Discharged home	38 (97.4)	44 (91.7)	0.25
Discharged home on post-operative day 1	23 (59.0)	33 (68.8)	0.34
Discharged home on or by post-operative day 2	32 (82.1)	38 (79.2)	0.74
Operative level			
L1–2	1 (2.6)	0	0.26
L2–3	2 (5.1)	3 (6.3)	0.82
L3–4	2 (5.1)	4 (8.3)	0.34
L4–5	19 (48.7)	25 (52.1)	0.75
L5-S1	15 (38.5)	16 (33.3)	0.62
Any perioperative complication	6 (15.4)	5 (10.4)	0.75
Return to OR for screw revision	1 (2.6)	0	0.26
Return to OR for hematoma evacuation	2 (5.1)	1 (2.1)	0.44
Wound infection	1 (2.6)	0	0.26
Nonsurgical or minor complication	2 (5.1)	4 (8.3)	0.34

LOS, length of stay; nominal data are presented as the number cases, *n* (%); continuous data are presented as mean ± SD; *p*-values <0.05 are considered significant. Nonsurgical or minor complication includes urinary retention after surgery, postoperative ileus, pneumonia, sepsis, and/or requirement for blood transfusion.

### 3.2 Screw accuracy

**Figure 2:**
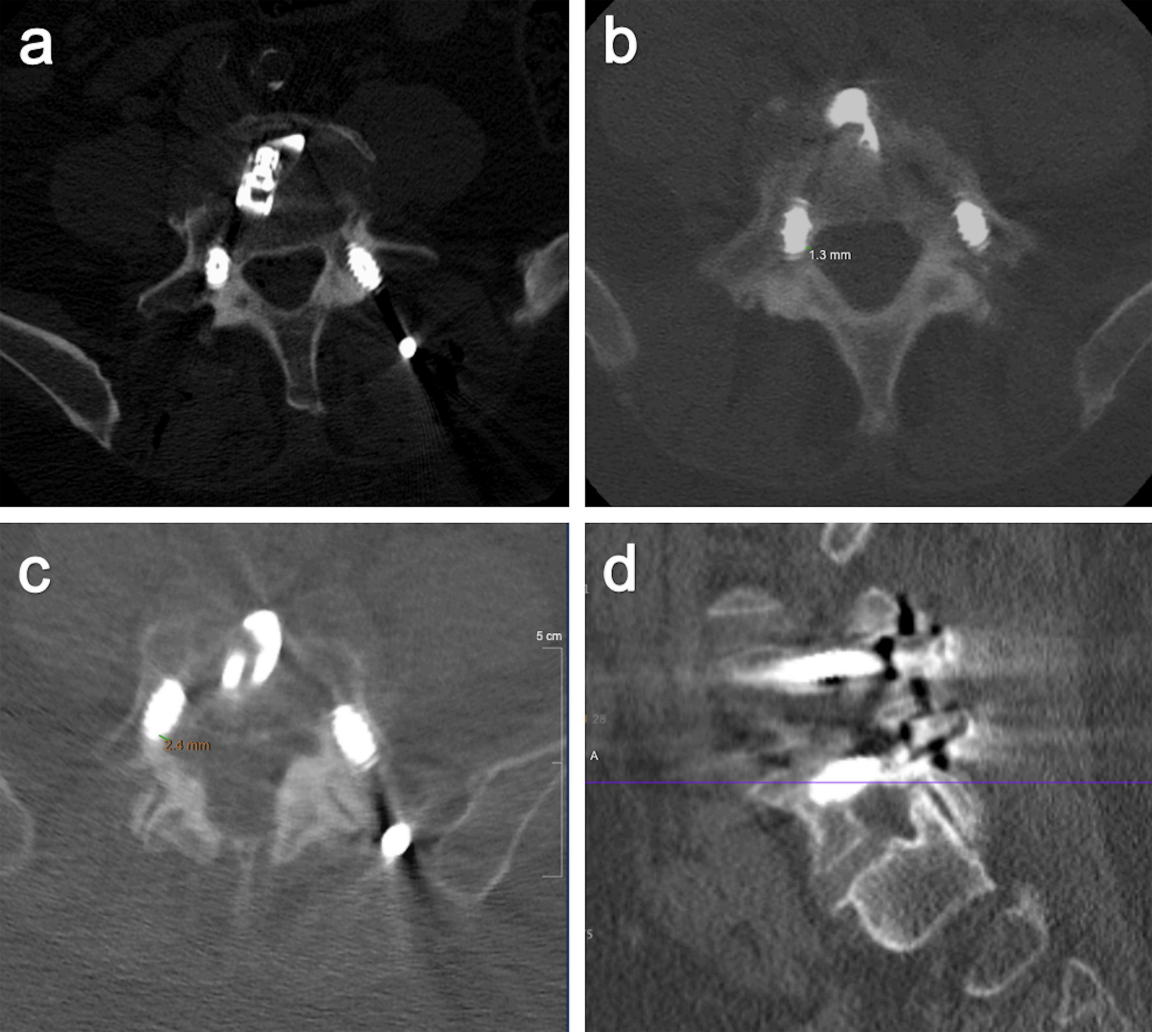
Postoperative computed tomography (CT) images of the L5 vertebral body of three separate patients demonstrating pedicle screw accuracy using the gertzbein and robbins classification system. Panel **(a)** demonstrates bilateral Grade A screw placement. Panel **(b)** shows right Grade B (<2 mm cortical breach) and left Grade A screw placement (no cortical breach). Panels **(c)** and **(d)** show a patient with a medial and inferior breach of the right L5 pedicle screw, classified as Grade C (<4 mm cortical breach) on axial **(c)** and sagittal **(d)** views. No Grade D or E cases are shown as there were none observed in this study.

A total of 348 screws were graded, including 156 in the FA group and 192 in the RA group (Table 3). The RA group had statistically significantly better screw placement compared to the FA group (*p* < 0.001). Eighty-nine (57%) screws in the FA group and 175 (91%) in the RA group were found to have grade A pedicle screw accuracy. Sixty-four (41%) screws in the FA group and 17 (9%) in the RA group were found to have grade B pedicle screw accuracy. FA had 3 (2%) screws with a grade C rating, while RA had none. There was no significant difference in screws with acceptable (grade A + grade B) placement between the FA (98%) and RA (100%) groups. Patients in the RA group had a significantly higher proportion of all four pedicle screws being graded as having grade A (FA: *n* = 3/39, 7.7% vs. RA: *n* = 33/48, 69% [*p* < 0.0001]). One patient in the FA group required revision surgery for screw repositioning while none were required in the RA group. Figure 2 depicts examples of Grade A, B, and C pedicle screw accuracy scores from patients in this study.

**Table 3:** Screw accuracy based on the Gertzbein and Robbins classification.

Screws (*N* = 348)	Fluoroscopy-assisted (FA)	Robot-assisted (RA)	*p*-value
Gertzbein and Robbins grade (*N* = 348)	*n* = 156	*n* = 192	
Screw accuracy by grade (per screw)			<0.001
Grade A	89 (57)	175 (91)	
Grade B	64 (41)	17 (9)	
Grade A + B (acceptable accuracy)	153 (98)	192 (100)	
Grade C	3 (2)	0	
Grade D	0	0	
Grade E	0	0	

Patient (*N* = 87)	*n* = 39	*n* = 48	
Screw accuracy by grade (per patient)			<0.001
All grade A	3 (7.7)	33 (69)	
All grade A or B (acceptable accuracy)	36 (92)	48 (100)	
Any grade C	3 (7.7)	0	

Screw accuracy is based on the Gertzbein and Robbins classification system. Grades A and B are considered acceptable while grades C, D, E are considered misplaced. The number of screws meeting the criteria for a given screw accuracy grade are presented as *n* (%). *P*-values <0.05 are considered significant.

### 3.3 Preoperative, inpatient, and long-term postoperative opioid consumption

Total MME consumption was significantly higher in the FA group on POD 0–1 (110.5 ± 63.0 vs. 69.2 ± 54.7, *p* = 0.002), while not on POD 2 (59.2 ± 61.9 vs. 38.8 ± 39.7, *p* = 0.15). The unadjusted mean daily MME consumption for total LOS was significantly higher in the FA group (52.1 ± 32.0 vs. 31.8 ± 24.3, *p* = 0.0015). In multivariable linear regression adjusting for age, BMI, and opioid naïve status, the RA approach was independently associated with significantly lower inpatient opioid use on POD0–1 (*β* = −37.1; 95% CI, −63.6 to −10.6; *p* = 0.007) and average daily MME for total LOS (*β* = −17.1; 95% CI, −29.6 to −4.7; *p* = 0.008). In multivariable linear regression models that adjusted for preoperative ODI and VAS scores, no statistically significant association with inpatient MME consumption was detected.

The number of patients taking opioids within 30 days of their elective operation was similar in the two groups [FA: 17/39 (43.6%) vs. RA: 17/48 (35.4%), *p* = 0.51]. Long-term postoperative opioid use was not significantly different between FA and RA groups at 3-, 6-, and 12-month postoperative intervals (*p* = 0.92). These are listed in Table 4.

**Table 4:** Preoperative, inpatient, and long-term postoperative opioid use.

(*N* = 87)	Fluoroscopy-assisted (FA) (*n* = 39)	Robot-assisted (RA) (*n* = 48)	*p*-value
Opioid users within 30 days of surgery	17 (43.6)	17 (35)	0.51
Inpatient opioid consumption			
MME on postoperative day 0 and 1	110.5 ± 63.0 (*n* = 36)	69.2 ± 54.7 (*n* = 47)	0.002
MME on postoperative day 2	59.2 ± 61.9 (*n* = 13)	38.8 ± 39.7 (*n* = 16)	0.15
Mean daily MME per day for total LOS	52.1 ± 32.0 (*n* = 36)	31.8 ± 24.3 (*n* = 47)	0.0015
Long-term postoperative opioid use			
Three-months	19 (49)	17 (35)	0.28
Six-months	17 (44)	12 (25)	0.11
Twelve-months	16 (41)	14 (29)	0.27

MME, morphine milligram equivalents; LOS, length of stay. Nominal data are presented as the number cases, *n* (%), that indicate “yes”; (%); continuous data are presented as mean ± SD; *p*-value <0.05 considered significant. Four patients (3 FA, 1 RA) did not have documented inpatient MME use in the medication record summary and were excluded from analysis.

### 3.4 Patient reported outcomes

PROs were available for all patients at either 6- or 12-month follow-up. Twenty patients who received FA treatment and 33 patients who received RA treatment completed all 6-month PROs (ODI, VAS leg, and VAS back). Thirty-two patients from the FA treatment group completed 12-month ODI and VAS leg pain, and 31 patients completed VAS back pain PROs. Thirty-eight patients from the RA treatment group completed 12-month ODI, and 41 patients completed 12-months VAS back and leg pain PROs.

Both groups achieved statistically significant improvement in ODI, VAS back, and VAS leg scores at 6- and 12-months, compared to preoperative scores. The change in ODI, VAS back, or VAS leg from baseline to 6-months and baseline to 12-months postoperatively was not significantly different between RA and FA groups, nor was the proportion of patients achieving MCID or meeting PASS criteria in groups. There was no significant difference in average patient satisfaction between RA (mean: 8.28 ± 2.12; 95% CI: 7.66–8.89) and FA (mean: 7.88 ± 2.77; 95% CI: 6.99–8.77) groups (*p* = 0.884). All patient-reported outcomes at 6- and 12-months for the FA and RA groups are listed in Table 5.

**Table 5:** Six- and 12-month patient reported outcome measures.

PROs	Fluoroscopy- assisted (FA)	95% CI	Robot- assisted (RA)	95% CI	*p*-value
ODI					
Pre-op	55.69 ± 13.23	51.30–59.88	49.75 ± 14.40	45.57–53.93	0.054
Δ at 6 mos	12.1 ± 4.24	3.26–20.93	20.18 ± 17.64	14.02–26.33	0.11
MCID or PASS at 6 mos	11 (55.0%)		24 (72.7%)		0.24
Δ at 12 mos	13.33 ± 26.94	2.68–24.0	20.36 ± 21.88	11.87–28.84	0.29
MCID or PASS by 12 mos	18 (56.3%)		26 (68.4%)		0.33
VAS back					
Pre-op	7.18 ± 2.47	6.38–7.98	6.17 ± 3.01	5.29–7.04	0.10
Δ at 6 mos	1.75 ± 2.92	0.39–3.12	2.39 ± 3.14	1.28–3.51	0.46
MCID or PASS at 6 mos	13 (65.0%)		25 (75.8%)		0.53
Δ at 12 mos	2.23 ± 4.12	0.57–3.9	2.35 ± 3.81	0.9–2.8	0.92
MCID or PASS by 12 mos	21 (80.8%)		32 (78.1%)		0.76
VAS leg					
Pre-op	5.97 ± 3.35	4.89–7.06	5.13 ± 3.47	4.11–6.13	0.25
Δ at 6 mos	2.9 ± 3.88	1.1–3.69	2.93 ± 3.65	1.09–4.72	0.63
MCID or PASS at 6 mos	15 (75.0%)		25 (75.8%)		0.99
Δ at 12 mos	2.3 ± 4.5	0.27–3.59	1.93 ± 4.29	0.52–4.08	0.76
MCID or PASS by 12 mos	22 (68.8%)		33 (78.8%)		0.42

The change in patient reported outcome (PRO) measures between baseline to 6 months (mos) and baseline to 12 mos are presented as the mean ± SD and 95% confidence interval (CI). PROs assessed here include the Oswestry Disability Index (ODI), and Visual Analog Scale (VAS) for back and leg pain. PRO follow-up occurs in a window within 2 months before or after the follow-up interval. The number of patients that met Minimal Clinically Important Difference (MCID) or Patient Acceptable Symptom State (PASS) is represented as *n* (%). The MCID cutoffs used for ODI and VAS pain scores are 13 and 1, respectively. Twenty patients who received FA treatment and 33 patients who received RA treatment completed all 6-month PROs (ODI, VAS leg, and VAS back). Thirty-two patients from the FA treatment group completed 12-month ODI and VAS leg pain, and 31 patients completed VAS back pain PROs. Thirty-eight patients from the RA treatment group completed 12-month ODI, and 41 patients completed 12-months VAS back and leg pain PROs.

Multivariable linear regression models adjusting for preoperative VAS and ODI scores showed no significant difference in 6- or 12-month PROs between the RA and FA groups. Preoperative symptom severity was the strongest predictor of postoperative outcomes (*p* < 0.01). In multivariable logistic regression adjusting for preoperative scores, age, BMI, and opioid naïve status showed no significant difference in the odds of achieving MCID or meeting PASS between RA and FA groups for any PRO.

## 4 Discussion

We present a retrospective comparative cohort study of patients undergoing elective, single-level RA and FA MIS-TLIF by a single surgeon. Compared to FA, the RA approach resulted in more accurate pedicle screw placement and lower inpatient MME consumption, with similar long-term opioid use, PRO improvement, and rates of achieving MCID or meeting PASS criteria.

Since the introduction of spinal robotic systems in 2004, a growing body of literature has demonstrated favorable safety and efficiency profiles (20–24). Studies on this topic heretofore have reported improved screw accuracy, along with reduced radiation exposure in RA compared to traditional FA techniques (8, 17, 25–27). The first study of the Mazor X Stealth device in 2019 reported 98.7% accuracy rate for grade I placement on the Ravi scale (23). One retrospective cohort study of patients undergoing RA MIS-TLIF for spondylolisthesis reported that no patients required revision surgery for screw malposition (28). Another study evaluating RA lumbar fusions reported an intraoperative complication rate of 3.4% related to the robot including intraoperative exchange of screw (0.9%), robot abandonment (2.5%), and return to the operating room for screw exchange (1.3%) (29). In the present study, all RA screws were acceptable (grade A or B), with 91% achieving grade A accuracy, compared to 57% in the FA group. However, the overall rate of acceptable screw placement (grade A or B) was largely similar in both groups (98% in FA group vs. 100% in RA group). Notably, only one patient of the FA group required revision surgery to reposition pedicle screw.

Existing studies evaluating PROs between RA and FA approaches have largely demonstrated similar long-term outcomes (9, 17, 27), though data on opioid utilization remain limited (30). In this study, RA was associated with lower inpatient MME consumption during the immediate postoperative period (POD 0–1) and across total LOS. No difference was observed on POD 2, likely due to early discharge in most patients (59% FA, 67% RA discharged on POD 1; Table 2), resulting in smaller sample sizes. Despite reduced inpatient MME consumption in the RA group, long-term opioid use at 3, 6, and 12 months was similar between groups. Both cohorts demonstrated significant improvements in ODI and VAS scores at 6 and 12 months (*p* < 0.05), with no between-group differences in PRO improvement or rates of achieving MCID or PASS on unadjusted univariate or multivariable regression analysis. Given the increasing focus on opioid stewardship in spine surgery (12, 31), these findings are clinically relevant.

The mechanism underlying reduced immediate postoperative MME consumption in the RA group remains unclear. Both techniques are percutaneous; however, FA techniques may experience greater tissue manipulation with Jamshidi needle use and potentially have larger incisions due to a lack of navigation. In contrast, the RA approach may allow for more precise incision planning and trajectory optimization, although this cannot be directly measured in the present study. To our knowledge, immediate postoperative MME consumption has not been previously studied in this population.

One potential criticism of the RA approach is the additional time required to set up and register the robotic device and instruments. A prior comparison of MIS-TLIF done with the Mazor robot found an added average of 42.25 ± 28.35 min compared to traditional FA approach (32). Furthermore, a prior study published by our group using the Mazor X device demonstrated significantly longer anesthesia times with RA approach (5 min longer per screw, *p* = 0.009) in single- and multi-level lumbar fusion surgeries with multiple surgeons (17). In the present study, of only single-level MIS-TLIF by a single surgeon, anesthesia times were an average of 8 min longer in the RA group (mean 190 vs. 182 min), however, not reaching significance (*p* = 0.24). Operative times between RA and FA groups were similar, and intraoperative fluoroscopy doses were significantly lower in the RA group, both findings consistent with other retrospective studies of this kind (8).

There are several limitations to this study. First, given the retrospective nature of the study, our results are susceptible to methodological bias. Second, to assess screw accuracy, patients were required to have a postoperative CT scan. While most patient in the RA cohort underwent postoperative CT scans during hospital admission, only 39 of 54 potential patients in the FA group had a postoperative CT. This resulted in the exclusion of several patients from the study and reduced number of patients that could be included (Figure 1). Thus, it is possible that the reported rate of screw accuracy in the FA group would have been either higher or lower if more CT scans were available. Furthermore, although the validated Gertzbein and Robbins classification system was used to assess screw accuracy, we acknowledge the potential for observer-related variability. Third, both cohorts exhibited a low rate of PRO responders, which likely led to an underestimation of the number of patients meeting MCID and/or PASS. Fourth, long term opioid use was determined by opioid prescriptions dispensed at 3-, 6-, and 12-month intervals following surgery, though it remains unknown what the interval and amount patients truly consumed at home. We acknowledge that this represents an estimation and is not a precise summary of long-term opioid use. Fifth, although limited to single-level MIS-TLIF performed by a single surgeon over a relatively short time, inclusion of index and revision cases, patient selection, small sample size, variable indications, decompression when indicated, and surgeon learning curve may confound outcomes and limit generalizability. It is also possible that changes in anesthesia administration and or inpatient hospital protocols could have affected aspects of inpatient care and introduced unknown confounders. Sixth, while we feel the FA comparator group is an adequate control, the overlap period when both techniques were utilized at the discretion of the senior author during the first year of RA approach implementation may be considered a limitation and potential confounder. Moreover, this study spans several years and we acknowledge the possibility that changes in patients care could have potentially affected control of postoperative pain and timely discharge could have been affected by differences in nursing or staff practices. Finally, as this was a retrospective study, a formal *a priori* power calculation was not performed; the sample size reflects all consecutive eligible patients during the study period. For non-significant comparisons, the possibility that the study was underpowered to detect smaller but clinically meaningful differences cannot be excluded, and 95% confidence intervals are provided to allow readers to assess the precision of effect estimate. The logistic models in particular should be interpreted with caution given the sample size constraints.

## 5 Conclusion

RA MIS-TLIF had improved pedicle screw accuracy and reduced early postoperative opioid consumption compared to FA techniques; however, these advantages did not translate into reduced long-term opioid use or improved patient-reported outcomes.

## Data Availability

The raw data supporting the conclusions of this article will be made available by the authors, upon a reasonable request.
